# Data-Rich But Integration-Poor Human Trials: Conceptual Framework for Closing the AI Transformation Gap in Dietary Intervention and Cosmetic Human Trials

**DOI:** 10.2196/103706

**Published:** 2026-10-23

**Authors:** Sungwon Lee, SooYeon Lee, Sunghee Lee, Hongseok Yoo, Jung Min Cho

**Affiliations:** 1 Department of Software, Daegu Haany University, Gyeongsan-si, Gyeongsangbuk-do, Republic of Korea; 2 Department of Cosmetics, Daegu Haany University, Gyeongsan-si, Gyeongsangbuk-do, Republic of Korea; 3 Department of Computer Science, Kyungpook National University, Daegu, Republic of Korea; 4 R&D Center, NUDIX, Gumi-si, Gyeongsangbuk-do, Republic of Korea; 5 School of Software, Kyungwoon University, Gumi-si, Gyeongsangbuk-do, Republic of Korea; 6 Functional Food Institute, Daegu Haany University, 1, Hanuidae-ro, Gyeongsan-si, Gyeongsangbuk-do, 38610, Republic of Korea, 82 1094958061

**Keywords:** AI, clinical trials, data integration, dietary supplements, cosmetics

## Abstract

Human trials in dietary and cosmetic domains generate large volumes of multimodal data but remain limited in their ability to produce integrated and actionable insights. Data such as intake records, physiological biomarkers, imaging outputs, and user-reported outcomes are typically collected through disconnected systems and analyzed independently, restricting the ability to establish time-resolved relationships between exposure and response. In this viewpoint, we define this limitation as the AI transformation (AX) gap, representing a structural disconnect between data generation and actionable knowledge in human trials. Drawing on evidence from real-world industry demand, we highlight how this gap is consistently observed across dietary intervention trials, cosmetic human trials, and underlying data systems. We propose a conceptual AX-driven framework that integrates multimodal data acquisition, continuously aligns intervention exposure with corresponding physiological, phenotypic, and participant-reported responses across time, and supports centralized analytical processing. The framework is intended to provide a unified perspective for transforming fragmented human-trial data into integrated, interpretable, and actionable knowledge. Although the proposed framework has not yet been empirically validated, it offers a conceptual foundation for future development and evaluation of integrated data platforms in dietary intervention and cosmetic human trials. Closing the AX gap may facilitate the transition from fragmented data collection toward human trials in which multimodal exposure and response data are continuously linked across time to support more integrated and individualized interpretation in research and product development.

## Introduction

### The Hidden Limitation of Human Trials

Human trials have long been regarded as the gold standard for evaluating the efficacy and safety of interventions in human populations [1]. In pharmaceutical and medical research, these trials have undergone substantial methodological and infrastructural evolution over recent decades. The adoption of standardized protocols (International Council for Harmonisation—Good Clinical Practice), regulatory oversight, centralized data management systems, and advanced statistical methodologies has markedly improved data quality, reproducibility, and clinical interpretability [2]. Consequently, pharmaceutical trials are now capable of generating robust and actionable evidence that informs regulatory approval and clinical decision-making [3].

In contrast, dietary intervention trials and cosmetic human trials have evolved along a different trajectory. Although the number of studies in these domains has rapidly increased—driven by rising demand for functional foods, personalized nutrition [4], and targeted skincare solutions [5]—their underlying data infrastructures have not advanced at a comparable pace. This imbalance has created a critical but underrecognized limitation: while these trials generate large volumes of diverse data, their ability to integrate and interpret such data remains constrained.

A key characteristic of both dietary intervention trials and cosmetic human trials is the inherent heterogeneity of collected data. In dietary intervention settings, researchers must simultaneously capture intake information (eg, food records and supplement use), physiological biomarkers (eg, blood glucose and lipid profiles), and behavioral or lifestyle variables (eg, physical activity and sleep patterns). For example, a participant may record daily supplement intake via a dietary log, undergo blood sampling at scheduled clinic visits, and wear a device that continuously tracks heart rate and activity levels. These data are typically stored in separate systems, often without standardized formats or integration protocols, making it difficult to analyze them collectively [6]. In addition, a similar complexity arises in cosmetic human trials, where outcomes are assessed through a combination of subjective and objective measures [7]. Participants may report perceived changes in skin condition through questionnaires, while researchers simultaneously collect high-resolution facial images and device-based measurements such as skin hydration, elasticity, or sebum levels. Each data type is generated through distinct acquisition tools and processed using independent pipelines. As a result, relationships between subjective perception and objective measurements—such as whether reported improvements correspond to measurable changes in skin properties—are rarely explored in a systematic manner [8].

Beyond data heterogeneity, the temporal nature of exposure and response further complicates the analysis. Unlike pharmaceutical trials, which often rely on clearly defined dosing schedules and end points, dietary and cosmetic interventions involve continuous and context-dependent exposures. Nutrient intake varies daily; product usage may be inconsistent; and responses are influenced by environmental conditions and individual variability [9]. However, most studies lack mechanisms to align time-resolved exposure data with corresponding biological or phenotypic outcomes. For instance, fluctuations in daily intake are rarely linked to short-term physiological responses [10], and repeated cosmetic measurements are often analyzed without considering temporal trajectories. This limits the ability to capture dynamic patterns and obscures individualized responses.

In addition, the operational structure of many such trials remains reliant on manual or semidigital workflows. Data are frequently entered from paper-based records or transcribed from multiple devices, introducing opportunities for error and inconsistency. Quality-control procedures are often retrospective rather than real-time, and standardization across datasets is limited. These constraints not only reduce data reliability but also hinder downstream analytical processes, particularly when integrating data across modalities and time points. Collectively, these challenges indicate that the primary bottleneck in dietary intervention trials and cosmetic human trials is not the absence of data but the inability to transform heterogeneous, time-dependent data into coherent and actionable insights. Despite being inherently data-rich, these studies often fail to produce reproducible, interpretable, and translatable outcomes.

We define this overarching limitation as the AI transformation (AX) gap: the disconnect between data generation and actionable knowledge in human trials. Addressing this gap requires more than incremental improvements in data collection; it demands a fundamental reconfiguration of how data are acquired, aligned, and analyzed. In particular, domains such as dietary intervention and cosmetic human trials—where multimodal, time-dependent, and user-driven data are central—require integrated frameworks capable of supporting continuous data linkage and interpretation.

The concept of digital transformation (DX) has long been used to describe the adoption of digital technologies for improving operational processes, organizational efficiency, and service delivery across health care and industry. However, as digital infrastructures have matured, the central challenge has shifted from digitizing data to generating actionable knowledge from increasingly heterogeneous and multimodal datasets. In this context, we propose the concept of AX, which extends beyond conventional DX by emphasizing the transformation of fragmented human-trial data into integrated, interpretable, and actionable knowledge through temporal alignment, multimodal data integration, and advanced analytical processes. Rather than representing another stage of digitalization, AX addresses the next methodological challenge of converting data-rich human trials into insight-driven research platforms. Accordingly, this viewpoint proposes an AX-driven perspective on human trials, developed through a multidisciplinary collaboration spanning clinical nutrition, cosmetic science, data systems, and digital health technologies.

### Why the AX Gap Matters

The domain-specific challenges, real-world implementation needs, and expected implications of the proposed AX framework are summarized in Table 1. The observations presented in this section are derived from two complementary sources: (1) published literature describing current technological and industrial trends in dietary interventions and cosmetic human trials, and (2) the authors’ direct experience obtained through industry-academic collaborative projects, technical consultations, and government-funded proposal development. Across sectors—including functional food development, cosmetic technology, and digital health platforms—similar patterns emerge, indicating a shared constraint in how human trial data are captured, managed, and translated into usable outcomes [11,12].

**Table 1.** Domain-specific challenges, implementation requirements, and expected implications of AI transformation (AX) in dietary intervention trials and cosmetic human trials.

Domain	Current challenges/limitations	Real-world needs	AX implementation	Expected implications
Dietary intervention trials	Limited temporal linkage between intake and physiological responses; mismatch between continuous exposure and intermittent biomarker measurements; fragmented analytical workflows	Integration of intake, biomarkers, and wearable signals; improved compliance monitoring; exposure-response evaluation	Continuous time-series linkage of intake and physiological responses; multimodal integration of dietary records, biomarkers, and wearable data	Improved dose-response analysis, personalized nutrition, and dynamic monitoring
Cosmetic human trials	Separation of subjective and objective assessments, heterogeneous imaging and sensor data, and limited longitudinal integration	Unified management of questionnaires, imaging, and skin bioparameters; validation of perceived efficacy	Integration of participant-reported outcomes, imaging, skin bioparameters, and product-use data within a common analytical framework	Cross-modal efficacy evaluation, individualized skincare, and longitudinal skin-response analysis
Data systems and platforms	Fragmented storage, limited interoperability, manual workflows, and lack of temporal alignment and automated analytics	Centralized data platform; standardized data structures, real-time quality control, and decision-support tools	Standardized acquisition, quality control, temporal alignment, centralized analytical processing, and AI-assisted interpretation	Scalable human-trial platform, reproducible analysis, decision support, and actionable knowledge generation

Although no published reports have yet quantified the demand for AX-driven human trial platforms specifically, the rapid expansion of decentralized clinical trials (DCTs) provides strong indirect evidence of increasing demand for integrated digital infrastructure capable of supporting multimodal data acquisition, longitudinal monitoring, and centralized analytical workflows. Recent regulatory initiatives, scientific evidence, and market analyses collectively indicate a rapidly increasing demand for digitally integrated human trial infrastructure. In 2023, the US Food and Drug Administration released guidance supporting DCTs, explicitly encouraging the use of digital health technologies for remote data acquisition, telemedicine-based follow-up, and decentralized participant management to modernize clinical trial conduct [13]. Consistent with this regulatory direction, a recent comprehensive analysis of 1370 DCTs registered in ClinicalTrials.gov [14] reported that DCT adoption has accelerated substantially since the COVID-19 pandemic. The number of newly registered decentralized trials increased from 102 in 2019 to 250 in 2023, while more than 50% of identified studies involved US collaborators and 42% were conducted internationally, demonstrating the rapid globalization of digitally enabled clinical research [15]. Similar trends are reflected in market forecasts by Fortune Business Insights, 2025, which estimate that the global DCTs market will expand from US $9.56 billion in 2025 to US $29.37 billion by 2034, corresponding to a compound annual growth rate of 13.2%, highlighting substantial industrial investment in digitally enabled clinical trial platforms.

Consistent with these broader trends, the authors have repeatedly encountered similar challenges through industry-academic collaborative projects and technical consultations related to dietary intervention and cosmetic human trials. These observations are summarized below. In dietary intervention trials, industry stakeholders frequently highlight the difficulty of linking intake data with physiological responses in a meaningful and timely manner [16]. In practice, intake information is typically collected through self-reported logs or mobile apps, while biological measurements—such as blood glucose, lipid profiles, or inflammatory markers—are obtained during scheduled clinical visits. For example, a participant may record daily supplement consumption over several weeks but only undergo biomarker assessment at baseline and at the end point. This mismatch in temporal resolution makes it challenging to determine how short-term variations in intake influence physiological responses. In addition, wearable sensor data (eg, activity levels or heart rate) are often stored on separate platforms and rarely integrated into the analytical workflow. As a result, compliance assessment, dose-response evaluation, and longitudinal pattern recognition remain limited, constraining the development of personalized nutrition strategies.

In cosmetic human trials, the integration challenge manifests in a different form, driven by the coexistence of heterogeneous data modalities [17]. These skin studies commonly involve the simultaneous collection of participant-reported outcomes, high-resolution skin images, and device-based measurements such as hydration, elasticity, and sebum levels. For instance, a participant may complete a questionnaire regarding perceived improvements in skin texture, while imaging systems capture wrinkle depth and pigmentation changes, and sensors quantify moisture levels at specific time points. However, each data type is typically processed using distinct software tools and stored in separate formats. This fragmented workflow prevents the systematic comparison of subjective perception with objective measurements, limiting the ability to validate efficacy claims or identify individualized response patterns. Furthermore, repeated measurements over time are often analyzed independently rather than as part of a continuous trajectory, reducing the interpretability of temporal changes.

From the perspective of data platform developers, the challenge extends beyond data acquisition to downstream use. Many existing systems are optimized for data storage and basic organization but lack advanced functionality required for integrated analysis [18,19]. For example, automated quality-control procedures—such as outlier detection or consistency checks across data streams—are often absent, and time-series alignment across modalities is rarely implemented. Similarly, tools for generating real-time summaries, visualization dashboards, or predictive indicators remain limited. Consequently, even when large datasets are accumulated, their potential to inform decision-making in research, clinical evaluation, or product development remains underexploited.

Despite differences in application domains, these industry observations converge on a common structural limitation: human trial data are generated through multiple disconnected processes and lack a coherent framework for integration and interpretation. This recurring pattern suggests that the challenge is not rooted in individual technologies or study designs but in the absence of a unified system capable of linking diverse data streams across time and modality.

Taken together, real-world industry demand highlights a critical gap between data generation and data use in human trials. This gap is not due to insufficient data availability but due to a lack of mechanisms that can connect, align, and transform data into meaningful insights. These observations provide empirical support for the concept introduced in this study—the AI transformation (AX) gap—which represents a systemic barrier to the advancement of data-driven dietary intervention trials and cosmetic human trials.

## The AX Framework

### Defining and Positioning the AX Gap

The need for the term “AX gap” arises from a limitation not fully captured by data fragmentation, interoperability, or translational gaps alone. A trial may use digital data capture, interoperable systems, and multimodal datasets and yet still fail to determine how a specific exposure at a given time relates to subsequent responses measured across different modalities and time points. We define this unresolved disconnect between temporally structured exposure and multimodal response as the core of the AX gap. Thus, the added value of the AX concept is not simply to call for more integration but to place continuous exposure-response alignment at the center of the human-trial data life cycle and connect it to subsequent statistical, machine learning (ML), and AI-assisted interpretation.

The increasing adoption of digital technologies has led to the emergence of several closely related concepts, including DX, data interoperability, multimodal data integration, and AI-enabled analytics. Although these concepts have substantially advanced health care research and digital infrastructure, they primarily address different levels of the data ecosystem and therefore do not fully capture the specific challenges encountered in dietary intervention and cosmetic human trials. DX has traditionally focused on replacing conventional workflows with digital technologies to improve operational efficiency, connectivity, and service delivery [20]. Data interoperability emphasizes the seamless exchange of information between heterogeneous systems through standardized formats and communication protocols. Similarly, multimodal data integration seeks to combine heterogeneous data types, whereas AI-based analytical approaches focus on extracting patterns or generating predictions from available datasets. Collectively, these approaches contribute to the digitalization and use of biomedical data; however, they generally assume that integrated and well-structured datasets are already available.

Other established concepts in digital health similarly address specific aspects of the biomedical data ecosystem but differ in scope from the proposed AX framework. For example, the translational gap in real-world data research primarily refers to the difficulty of translating routinely collected health care data into clinically meaningful evidence. Common data models, such as the Observational Medical Outcomes Partnership, focus on harmonizing heterogeneous datasets into standardized database structures to facilitate interoperability and large-scale analyses. Similarly, the FAIR (findable, accessible, interoperable, and reusable) data principles provide guidelines for improving data stewardship and reusability, while multimodal sensor fusion emphasizes the technical integration of multiple sensing modalities to improve analytical performance. Digital twin frameworks further extend these approaches by constructing virtual representations of individuals or biological systems to simulate future health states. Although each of these concepts addresses an important component of digital health, they generally focus on data standardization, interoperability, simulation, or analytical performance after data have been collected. In contrast, the proposed AX framework specifically addresses the methodological challenge of continuously linking multimodal exposure and response data throughout the entire human-trial workflow, thereby enabling the transformation of fragmented longitudinal observations into actionable scientific knowledge.

The AX gap proposed in this study addresses a different and largely overlooked challenge. Rather than focusing on digitization, interoperability, or analytical modeling individually, AX emphasizes the transformation of fragmented human-trial data into integrated, temporally aligned, and interpretable knowledge. It should be noted that the proposed AX framework is broader than AI itself. The principal novelty of AX, therefore, does not lie in the application of AI itself but in integrating the entire human-trial data life cycle—from multimodal data acquisition and standardization to continuous temporal exposure–response alignment, analytical processing, and interpretation—within a unified conceptual framework. Several components of the framework—including multimodal data acquisition, data standardization, interoperability, quality control, and temporal alignment—can be implemented using conventional digital infrastructures and do not inherently require AI. Rather, AI represents one analytical component within the broader AX framework, where conventional statistical methods, ML, and AI-assisted analytics collectively contribute to transforming integrated human-trial data into actionable knowledge. Specifically, AX targets the structural disconnect between exposure data (eg, dietary intake or cosmetic product use) and multidimensional biological or phenotypic responses, which are often collected independently across different devices, platforms, and time points. This perspective extends beyond conventional data integration by emphasizing continuous exposure-response linkage, longitudinal harmonization, and knowledge generation within a unified analytical framework. Accordingly, AX should not be regarded as a replacement for DX or other established digital health approaches. Rather, it builds upon their complementary functions: DX provides the digital infrastructure; interoperability and common data models enable data exchange and harmonization; FAIR principles support data stewardship and reuse; multimodal data integration combines heterogeneous data sources; and DCTs extend data acquisition beyond conventional trial sites. The proposed AX framework connects these functions across the human-trial data life cycle, with its distinctive emphasis on continuous temporal alignment between exposure and multimodal responses and their subsequent transformation into interpretable knowledge. Table 2 summarizes how the proposed AX framework conceptually extends, rather than replaces, established digital health approaches.

**Table 2.** How AI transformation (AX) extends established digital health concepts.

Established concept	Extension provided by the AX framework
DX^a^	Extends digitalization of workflows toward integration of the entire human-trial data life cycle
Data interoperability	Extends technical data exchange toward the continuous linkage of exposure and response across systems and time
FAIR^b^ data principles	Extends data stewardship and reusability toward the longitudinal use of integrated data for interpretation
Multimodal data integration	Extends combination of heterogeneous modalities toward time-aligned exposure-response integration
DCTs^c^	Extends distributed and remote data acquisition toward the integration of decentralized data streams within a unified analytical workflow

a DX: digital transformation.

b FAIR: findable, accessible, interoperable, and reusable.

c DCT: decentralized clinical trial.

### Structural Characteristics of the AX Gap

To clarify the structural nature of the AX gap, Figure 1 illustrates prevailing data collection practices in human trials. In typical study settings, intake records, physiological measurements, and user-reported outcomes are captured through separate tools and workflows, often relying on paper-based documentation, stand-alone instruments, or unconnected digital systems. For example, dietary intake may be logged via mobile or paper diaries, blood biomarkers are processed within laboratory information systems, and wearable sensor outputs are stored on proprietary platforms. Because these data streams are not integrated within a centralized framework, establishing meaningful relationships between exposure and response becomes inherently difficult. Without a system capable of synchronizing data across time and individuals, multimodal inputs—including survey responses, biosignals, and imaging data—cannot be jointly analyzed in a coherent manner. As a result, large volumes of data remain structurally separated, constraining their capacity to generate interpretable and actionable insights.

**Figure 1.**
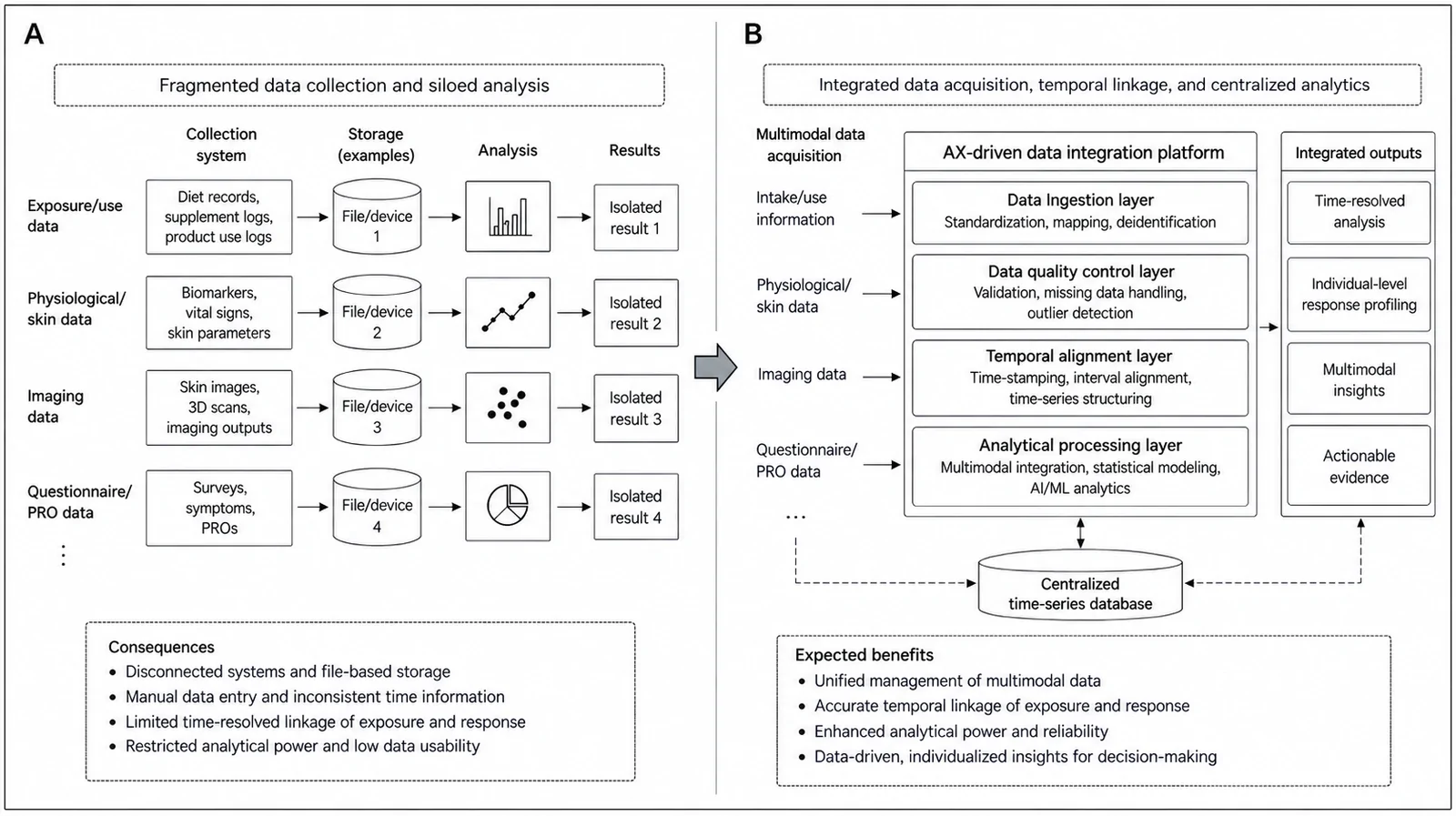
From fragmented to integrated human-trial workflows. This figure conceptually illustrates the transition from fragmented data collection to an integrated workflow capable of generating actionable knowledge from multimodal human-trial data. (A) Current state: multimodal data generated in dietary intervention trials and cosmetic human trials are collected through disconnected systems, stored in isolated files or devices, and analyzed independently. (B) Proposed state: an AX-driven integrated workflow enables centralized data acquisition, quality control, temporal alignment, and unified analytical processing. AX: AI transformation; ML: machine learning; PRO: participant-reported outcome.

The AX gap becomes especially evident in dietary intervention trials, which involve complex interactions among intake, physiology, and behavior over time. These studies typically require repeated measurements across multiple domains, including dietary records, blood-based biomarkers, wearable sensor outputs, and lifestyle variables. In practice, however, these measurements are collected at different temporal resolutions and stored in independent systems. For instance, daily intake records may be continuously recorded, whereas biomarkers such as glucose or lipid levels are assessed only at baseline and end-point visits. This discrepancy makes it difficult to determine how short-term fluctuations in intake influence physiological responses. Consequently, exposure and response are rarely linked within a continuous temporal framework. This disconnection limits the ability to evaluate dose-response relationships, monitor adherence, and identify individual-specific response patterns [21]. Clinically relevant biomarkers are frequently treated as isolated end points rather than as components of dynamic response trajectories. In addition, the logistical burden associated with repeated sampling, coupled with inconsistent data management practices, further restricts scalability and reproducibility in dietary intervention trials.

A related but distinct manifestation of the AX gap is observed in cosmetic human trials, where the primary challenge lies in integrating heterogeneous data across modalities [22]. These studies typically combine subjective assessments, imaging-based analyses, and device-derived measurements such as skin hydration, elasticity, and sebum levels [23,24]. For example, participant-reported perceptions of skin improvement may be collected via questionnaires, while high-resolution images are analyzed for wrinkle depth or pigmentation, and biophysical sensors quantify moisture or oil content. Each data type is generated and processed through separate acquisition and analytical pipelines, often in incompatible formats. As a result, integration across modalities remains limited, and analyses are commonly conducted within each data type independently. This prevents the systematic evaluation of relationships between subjective perception and objective measurements, limiting the ability to validate product efficacy or interpret individual variability. Furthermore, repeated measurements are often analyzed without temporal linkage, reducing the ability to identify longitudinal response patterns that are critical for personalized skincare strategies.

Despite differences in study design and measurement approaches, dietary intervention trials and cosmetic human trials share a common structural limitation: data are abundant but remain disconnected across modalities, time, and analytical processes. Consequently, the potential value of collected data is not fully realized, and resulting insights are often incomplete or inconsistent.

We define this recurring limitation as the AX gap—the structural disconnect between data generation and actionable knowledge in human trials. The AX gap is characterized by three core features: (1) fragmentation of multimodal data, (2) absence of time-resolved linkage between exposure and response, and (3) insufficient integration of analytical processes for interpretation and decision support. Addressing this gap requires not only technological advancement but also a fundamental redesign of how human trial data are structured, synchronized, and translated into meaningful outcomes.

### Operationalizing AX—A Unified Platform for Human Trials

The limitations described above are not isolated technical issues but reflect a systemic constraint in how human trials are currently designed and executed. Addressing the AX gap requires a transition from disconnected, modality-specific workflows toward an integrated framework capable of synchronizing multimodal data across time, domains, and analytical processes. Rather than representing a simple DX, AX involves restructuring the entire human-trial data life cycle—from acquisition and standardization to interpretation and decision support—within a unified platform.

As illustrated in Figure 2, the proposed AX-driven platform integrates multimodal data generated from both dietary intervention trials and cosmetic human trials into a common analytical framework. Although these 2 domains differ in exposure types and outcome measures, they share a common requirement for collecting, managing, and interpreting heterogeneous longitudinal data. In dietary intervention trials, data typically include dietary intake records, biometric measurements (eg, blood pressure and body composition), and wearable sensor outputs all managed within a unified system that supports continuous data acquisition and time-series analysis [25,26]. In cosmetic human trials, complementary data streams include product usage records, high-resolution skin images, device-derived skin parameters such as hydration, elasticity, and sebum levels, together with participant-reported assessments [27]. Conventionally, these datasets are acquired through independent devices and software systems, resulting in fragmented storage and limited interoperability.

**Figure 2.**
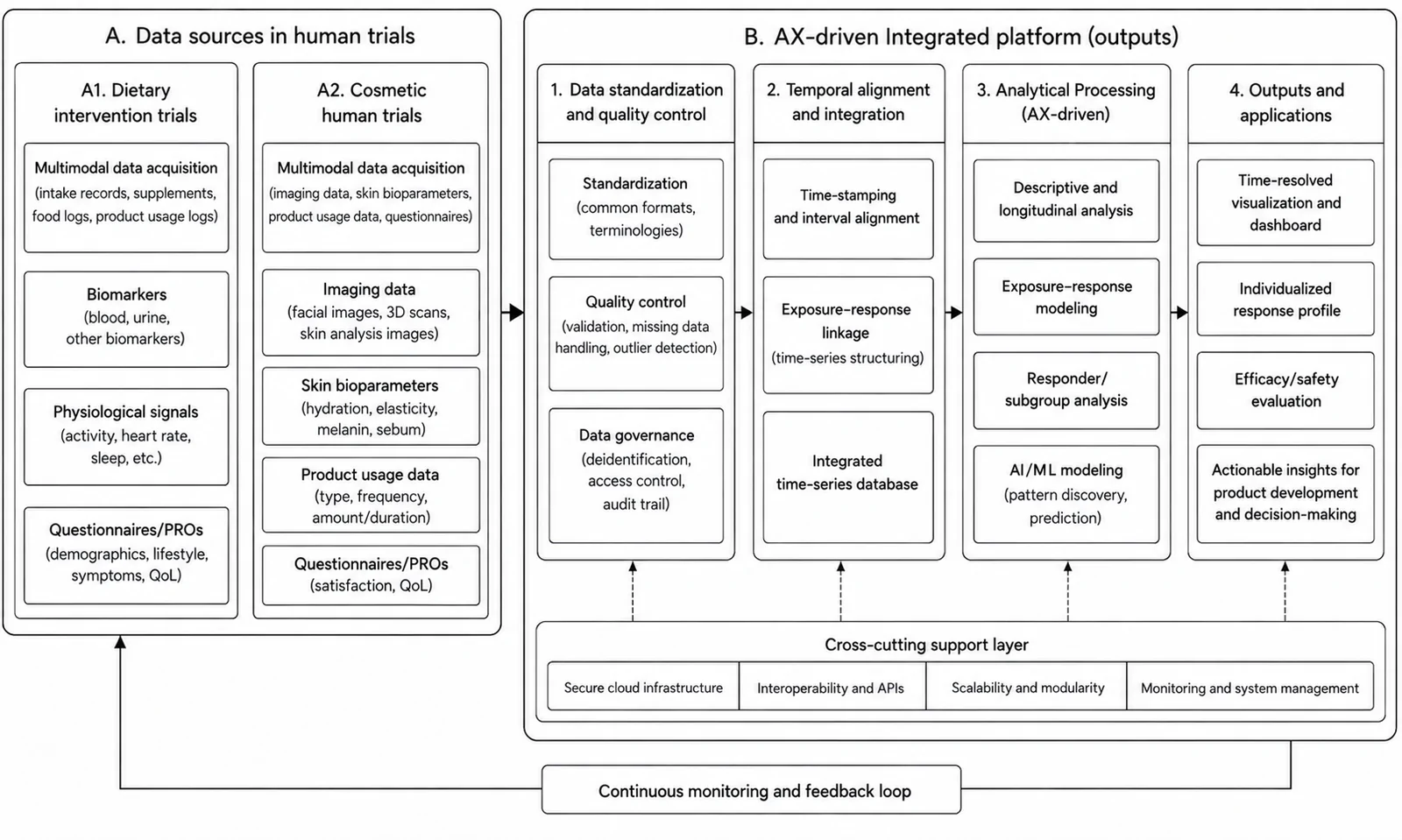
Unified AI transformation (AX)–driven platform integrating dietary intervention and cosmetic human trials. The platform distinguishes domain-specific data acquisition from the shared analytical framework, demonstrating how heterogeneous data are transformed into interpretable and actionable evidence through a unified AX-driven workflow. ML: machine learning; PRO: participant-reported outcome; QoL: quality of life.

The proposed platform addresses this limitation through a centralized workflow consisting of 5 sequential processes: multimodal data acquisition, data standardization and quality control, temporal alignment and integration, analytical processing, and generation of actionable outputs [18,28]. During data acquisition, domain-specific information from dietary intervention and cosmetic human trials is collected in standardized formats. These datasets subsequently undergo quality-control procedures, including data validation, missing-value handling, and outlier detection, followed by harmonization through common metadata structures and standardized terminologies. Temporal alignment then links exposure events—including dietary intake or cosmetic product application—with corresponding physiological, phenotypic, or participant-reported responses using shared timestamps and integrated time-series databases. This enables continuous exposure-response tracking that is rarely achievable in conventional human-trial workflows.

For example, participants in a dietary intervention trial may record daily supplement intake through a mobile app, while wearable devices continuously capture physical activity and heart rate, and laboratory biomarkers are obtained during scheduled clinic visits. Similarly, in cosmetic human trials, participant-reported outcomes, standardized facial images, and device-derived skin measurements are collected throughout the intervention period. Within the proposed AX framework, these heterogeneous datasets are automatically integrated, temporally aligned, and processed within a unified analytical environment, enabling investigators to evaluate exposure-response relationships, participant adherence, and individualized intervention responses across different human-trial domains.

Once integrated, the data are processed within a centralized analytical framework supporting descriptive and longitudinal analyses, exposure-response modeling, responder and subgroup identification, and AI-assisted pattern recognition. Robust analytical frameworks should also accommodate variability arising from different acquisition environments and data sources. Recent work has demonstrated that variations in real-world datasets can substantially influence model parameters and generalizability, highlighting the importance of designing analytical pipelines that remain robust across heterogeneous data conditions [29]. Within the proposed framework, conventional statistical analyses remain essential for hypothesis testing and effect estimation, whereas ML methods support prediction and subgroup identification. AI-assisted analytical approaches are intended to complement these methods by identifying complex multimodal relationships and generating higher-level interpretations from integrated datasets.

For example, conventional statistical analysis may provide an intervention effect estimate with a CI for a predefined biomarker; ML may generate an individual-level probability of response or classify participants into predicted responder groups based on longitudinal features; and AI-assisted multimodal analysis may identify cross-modal response patterns linking intervention exposure with physiological signals, imaging features, and participant-reported outcomes to generate integrated response profiles. Accordingly, AX should be understood as a comprehensive transformation framework, encompassing data acquisition, integration, and knowledge generation, rather than as an AI-only solution. The resulting outputs include time-resolved visualizations, individualized response profiles, efficacy and safety evaluations, and actionable evidence that can support both scientific interpretation and product development.

The platform is further supported by cross-cutting infrastructure components, including secure cloud-based data management, interoperability through standardized application programming interfaces, scalable system architecture, and continuous monitoring. Together, these components transform fragmented, domain-specific workflows into a unified and reproducible analytical ecosystem capable of supporting both dietary intervention trials and cosmetic human trials. By integrating heterogeneous data streams within a common temporal and analytical framework, the proposed AX platform enables a shift from isolated end point-based evaluation toward continuous, data-driven interpretation, thereby providing the operational foundation for closing the AX gap in human trials [30,31]. In addition, rather than functioning as passive storage environments, these systems act as active engines for insight generation, supporting both scientific investigation and real-world decision-making [32].

## Discussion

### Potential Implications of AX

The transition toward AX-driven systems has significant implications for the future of human trials, particularly in dietary intervention trials and cosmetic human trials. By addressing the structural limitations identified in previous sections, AX may enable a shift from fragmented data collection toward integrated, time-resolved, and insight-generating research frameworks.

As summarized in Table 3, this transformation represents a fundamental shift in how human-trial data are structured and used. Conventional approaches are characterized by disconnected datasets, manual data handling, and end point-based analysis, whereas AX-driven systems enable unified multimodal integration, continuous time-series alignment, and automated analytical processing. This transition has the potential to improve data quality and operational efficiency and expand the role of human trials from static evaluation systems toward dynamic platforms for knowledge generation and decision support.

While AX provides a unifying framework, its implementation is inherently domain-specific. In dietary intervention trials, the implications of AX are particularly evident in the ability to link intake with physiological responses over time. By integrating intake records, wearable biosignals, and clinical biomarkers within a unified framework, AX may enable the reconstruction of exposure-response trajectories at both the individual and population levels. This could facilitate more precise evaluation of dose-response relationships, improve adherence monitoring, and support the development of personalized nutrition strategies. Importantly, such capabilities move beyond conventional baseline-to-end point comparisons toward dynamic, longitudinal interpretations of intervention effects. In cosmetic human trials, AX-driven integration enables a comprehensive synthesis of heterogeneous data, including subjective assessments, imaging-based outputs, and device-derived measurements. By aligning these multimodal data streams within a shared analytical pipeline, researchers can explore cross-modal relationships, such as the concordance between perceived and measured skin improvements. This integration may support a more comprehensive evaluation of product efficacy and facilitate individualized response profiling, which may be relevant to personalized skincare applications.

**Table 3.** Transformational impact of AI transformation (AX)–driven integration in human trials.

Dimension	Conventional (fragmented system)	AX integrated system	Representative example	Practical impact
Data structure	Disconnected datasets (intake, biomarkers, and questionnaires)	Unified multimodal data integration	Daily dietary intake, blood biomarkers, wearable activity, and participant-reported outcomes stored within a single participant-level dataset	Enables comprehensive participant-level analysis
Temporal alignment	Cross-sectional or end point-based	Continuous time-series linkage	Daily supplement intake automatically aligned with weekly laboratory biomarkers and continuous wearable signals	Improves exposure-response tracking
Data acquisition	Manual entry and separate devices	Automated, synchronized data capture	Dietary records collected via mobile application, wearable data imported automatically, and laboratory results linked through participant ID	Reduces transcription errors and missing data
Analytical capability	Modality-specific analyses	Integrated multimodal analytics	Simultaneous analysis of dietary intake, blood biomarkers, skin images, and participant-reported outcomes within one analytical workflow	Supports multidimensional interpretation of intervention responses
Personalization	Population-level interpretation	Individual-level response modeling	Individual trajectories reconstructed from repeated intake records, physiological measurements, and skin assessments	Facilitates participant-specific evaluation
Data use	Data storage–focused	Knowledge generation and decision support	Real-time dashboards displaying adherence, physiological responses, and skin outcomes for investigators	Accelerates evidence generation and trial management
Monitoring and quality control	Retrospective data review	Continuous quality monitoring	Automated detection of missing records, abnormal biomarker values, and inconsistent timestamps during data acquisition	Improves data completeness and reliability
Trial output	Static end-point comparison	Dynamic longitudinal evaluation	Time-resolved visualization of biomarker trajectories and participant response profiles throughout the intervention	Supports adaptive interpretation and longitudinal evaluation

At a broader level, AX-driven integration has the potential to improve the reliability, scalability, and translational value of human trials. Automated data acquisition and centralized processing may reduce variability associated with manual workflows, while real-time monitoring and quality control may improve data integrity. Furthermore, structured and interpretable outputs—such as time-series visualizations and predictive indicators—could support more efficient decision-making in research and industry contexts. Importantly, not every component of the proposed framework requires AI implementation. Data integration, interoperability, and quality control rely primarily on established digital infrastructures, whereas AI contributes to advanced interpretation and decision support once harmonized datasets have been established. Collectively, these implications indicate that closing the AX gap is not merely a technical improvement but a critical step toward enabling the next generation of human trials. Without such transformation, the increasing volume and complexity of multimodal data will continue to outpace the capacity of conventional analytical frameworks, limiting both scientific insight and real-world applicability.

### Challenges and Future Considerations

Despite its potential advantages, the proposed AX framework should be regarded as a conceptual foundation rather than a fully validated implementation model. Several technical, ethical, and regulatory challenges must be addressed before AX-driven human-trial platforms can be widely adopted.

First, successful implementation requires robust governance of multimodal human-trial data. Continuous acquisition of dietary intake, wearable biosignals, physiological measurements, imaging data, and participant-reported outcomes inevitably raises important considerations regarding informed consent, participant privacy, data ownership, and cybersecurity. Because these data are often collected longitudinally and across multiple devices, clear governance frameworks are essential to ensure secure data management while maintaining participant trust.

Second, practical implementation depends on interoperability among heterogeneous data sources. Human trials frequently use instruments from different manufacturers, which generate data in incompatible formats. Therefore, establishing standardized data structures and communication protocols represents a prerequisite for integrating multimodal datasets within a unified platform. In addition, the financial costs associated with sensor infrastructure, cloud-based platforms, and long-term data management may limit implementation, particularly in small-scale academic or industry-sponsored studies.

Third, the implementation of AX-driven analytical frameworks should be accompanied by appropriate methodological and ethical safeguards. Although AI can facilitate the identification of complex multimodal relationships, AI-assisted outputs should complement rather than replace conventional statistical analyses and expert interpretation. Particular attention should be given to algorithmic bias, model transparency, and clinical accountability to ensure that generated insights are reliable, interpretable, and applicable across diverse populations. Furthermore, actionable insights derived from AX platforms should be supported by appropriate statistical validation and human oversight before being incorporated into scientific conclusions or decision-making processes.

Finally, the proposed framework has not yet been prospectively or empirically validated in real-world human trials, and, therefore, its anticipated benefits—including improved data reliability, personalized interpretation, and actionable knowledge generation—should be regarded as conceptual rather than demonstrated outcomes. Future studies should systematically evaluate the feasibility, usability, interoperability, and effectiveness of the proposed framework through prospective implementation in dietary intervention and cosmetic human trials. In particular, multicenter validation studies are needed to determine whether AX-driven platforms can improve data quality, analytical performance, workflow efficiency, and decision-making compared with conventional human-trial workflows. Furthermore, standardized implementation protocols, robust governance frameworks, and regulatory acceptance will be essential before the proposed framework can be translated into routine research practice.

## Conclusion

Human trials in dietary interventions and cosmetics are at a critical inflection point. While the capacity to generate diverse and high-volume data has expanded rapidly, the ability to integrate and interpret that data has not kept pace. This imbalance has resulted in a persistent AX gap, where data remain abundant but insufficiently translated into meaningful insights. Addressing this gap requires a fundamental transition from fragmented data collection toward integrated, time-aligned, and analytically driven systems. The principal conceptual contribution of the AX framework is the integration of fragmented multimodal data with continuous temporal exposure-response alignment and AI-assisted interpretation across the human-trial data life cycle.

As demonstrated throughout this viewpoint, AX-driven frameworks may provide a pathway to support this transformation by connecting multimodal data streams, enabling time-series analysis, and supporting integrated interpretation. Importantly, the AX gap is not a theoretical construct but a practical limitation already observed across multiple domains of human trials. Its impact is evident in the difficulty of linking exposure to response, the underuse of multimodal data, and the limited scalability of current study designs. Without resolving these structural issues, human trials will remain constrained in their ability to deliver reproducible, personalized, and actionable outcomes. Closing the AX gap may contribute to redefining the role of human trials from passive data collection exercises to more integrated, insight-generating systems that support precision-based decision-making. Such a transition may help advance scientific understanding and bridge the gap between research and real-world applications in dietary intervention and cosmetic human trials.

## Abbreviations

AX: AI transformation
DCT: decentralized clinical trial
DX: digital transformation
FAIR: findable, accessible, interoperable, and reusable
ML: machine learning
